# A Complete EGSE Solution for the SpaceWire and SpaceFibre Protocol Based on the PXI Industry Standard †

**DOI:** 10.3390/s19225013

**Published:** 2019-11-16

**Authors:** Luca Dello Sterpaio, Antonino Marino, Pietro Nannipieri, Gianmarco Dinelli, Daniele Davalle, Luca Fanucci

**Affiliations:** 1 Department Information Engineering, University of Pisa, 56122 Pisa (PI), Italy; luca.fanucci@unipi.it; 2 Space Division, IngeniArs S.r.l., 56121 Pisa (PI), Italy; daniele.davalle@ingeniars.com

**Keywords:** SpaceFibre, SpaceWire, instrumentation, test equipment, EGSE, PXI, LabVIEW, FPGA, field programmable gate arrays, spacecraft, communications, serial communications

## Abstract

This article presents a complete test equipment for the promising on-board serial high-speed SpaceFibre protocol, published by the European Committee for Space Standardization. SpaceFibre and SpaceWire are standard communication protocols for the latest technology sensor devices intended for on-board satellites and spacecrafts in general, especially for sensors based on image acquisition, such as scanning radiometers or star-tracking devices. The new design aims to provide the enabling tools to the scientific community and the space industry in order to promote the adoption of open standards in space on-board communications for current- and future-generation spacecraft missions. It is the first instrument expressly designed for LabVIEW users, and it offers tools and advanced features for the test and development of new SpaceFibre devices. In addition, it supports the previous SpaceWire standard and cross-communications. Thanks to novel cutting-edge design methods, the system complex architecture can be implemented on natively supported LabVIEW programmable devices. The presented system is highly customizable in terms of interface support and is provided with a companion LabVIEW application and LabVIEW Application Programming Interface (API) for user custom automated test-chains. It offers real-time capabilities and supports data rates up to 6.25 Gbps.The proposed solutions is then fairly compared with other currently available SpaceFibre test equipment. Its comprehensiveness and modularity make it suitable for either on-board device developments or spacecraft system integrations.

## 1. Introduction

This work is an in-depth discussion of the design and implementation of a novel test equipment for the SpaceFibre and SpaceWire protocols, the first natively supported and expressly designed protocols for the LabVIEW framework. Preliminary results of this work were presented at the 2019 IEEE International Workshop on Metrology for AeroSpace (MetroAeroSpace) [1]. If compared to those preliminary results, in this article, the system architecture is presented comprehensively and in depth, along with the innovative and cutting-edge methodologies for its implementation; also, the proposed system is compared with other currently available SpaceFibre instruments to provide a fair analysis.

### 1.1. Background Knowledge

According to [2], an on-board communication system denotes any on-board architecture that permits performing information transmission on-board a spacecraft. It can be implemented with different technologies, physical architectures, and redundancy schema. Such a communication system is currently demanded to withstand substantial data rates, mainly due to the usage of image based sensors: Earth observation missions are often equipped with imager sensors, Synthetic Aperture Radars (SARs), and other sensing payloads which, can require a large bandwidth in the order of one or even several Gbps to perform the routing of payloads related to data traffic throughout the rest of the spacecraft (data storage, telemetry transmitter, etc.).

SpaceWire is a communication protocol developed by the University of Dundee [3] for the European Space Agency (ESA) and standardized by ECSS in 2003 with a revision of the standard released in 2019 [4]. It has been designed to improve the on-board communication line from different points of view: it is a low error rate line, with low resource utilization (5–8 Kgates). The communication line is full duplex. SpaceWire is able to reach a data rate up to 400 Mbps (with dedicated hardware) in on-board application, making it appropriate for some payload-to-platform communication lines. The SpaceWire network solution has been already adopted for payload data handling in several ESA, NASA, and the Japan Aerospace eXploration Agency (JAXA) missions, such as Rosetta, Mars-Express, Galileo, and many others, making it a highly reliable solution. It also implements fault tolerance mechanisms, which switch automatically from one link to another in the case of failure with very small loss of information: no error recovery feature is included. SpaceWire is not a deterministic protocol itself, also due to the presence of routers inside a SpaceWire network, which creates even greater time uncertainty. This is not always acceptable, especially when determinism is a key application requirement. This led to the release of an additional standard named SpaceWire-D, which implements deterministic features on top of the SpaceWire protocol. A wide set of solutions of codecs and routers is available on the market, both Application Specific Integrated Circuit (ASIC) and Field Programmable Gate Array (FPGA) [5]. At the time of writing, SpaceWire is still a hot topic in the space community as almost all the recent space missions are equipped with such protocols. Therefore, the protocol is still relevant from the test equipment point of view as well. In current missions, most of the data traffic is made of packets including readings of peripheral sensor payload devices. Platform devices generate traffic as well for control or house-keeping, often aggregating readings of various monitoring sensors (i.e., voltage, current, and temperature). However, a set of future missions is currently under design or under proposal. The use of new sophisticated imaging sensors, for example camera instruments capturing hyper-spectrum frames at very high resolution, will definitively require payload related data traffic bandwidth in the order of several Gbps. SpaceWire will not therefore be able to fulfill such requirements. For this reason, the European Space Agency started working with the University of Dundee and all the SpaceWire community on the evolution of the protocol: SpaceFibre.

SpaceFibre standardization by the European Committee for Space Standardisation (ECSS) [6] was completed in May 2019. It is a high speed communication protocol developed by the University of Dundee under ESA supervision [3].Compared to other proprietary solutions derived from information technology to address the data rate issue, SpaceFibre remains an open standard. It is a multi-lane protocol (up to 16 lanes can operate in parallel) able to provide data rates up to 6.25 Gbps per single lane, overcoming all previous limitations in terms of maximum achievable bandwidth. It can run both on copper cables and on optical medium. The innovative Quality of Service (QoS) mechanism implemented in the SpaceFibre standard provides concurrent bandwidth reservation, packet priority, and scheduling. Advanced Fault Detection, Isolation, and Recovery (FDIR) techniques are also included in the SpaceFibre protocol, improving system reliability. SpaceFibre supports multiple accesses with a virtual bus mechanism, named virtual channels. Error containment has been introduced in both virtual channels and frames, along with transparent recovery from transient errors, galvanic isolation, and “babbling idiot” protection. This relieves the upper layers to check the data integrity; thus, it reduces both development and system validation in terms of time and cost. In the literature, there are also reduced feature SpaceFibre interfaces, which benefit from the high performances of SpaceFibre in terms of bandwidth, with very low resource utilization [7] in exchange for non-critical (for the particular application) advanced features. Furthermore, SpaceFibre is backward compatible at the packet level with SpaceWire: this easily enables interconnection of SpaceWire devices into a SpaceFibre network, gaining the QoS and FDIR advantages of SpaceFibre. The SpaceFibre technology is getting more and more mature: not only single interfaces, but also routing switches [8] have been developed, together with higher level instruments such as a mixed SPW/SPFI network simulator [9].

SpaceFibre is an open standard. Being openly accessible is not a characteristic to be overlooked considering that aerospace is nevertheless a strategic sector. Indeed, other solutions exist on the market to address the data rate and time-determinism requirements of modern space missions, yet they are proprietary. TTEthernet [10] is a technology developed by TTTech. It is an extension of the Institute of Electrical and Electronics Engineering (IEEE) 802.3 standard, making TTEthernet fully compatible with the Ethernet standard. TTEthernet (TTE) implements along the best-effort Ethernet the time triggered communication paradigm of the SAE AS6802 standard and the reserved-bandwidth protocol of ARING664p7. This makes it perfectly suitable for safety critical applications like space missions. TTE is a noteworthy example of Ethernet based solutions, derived from Gigabit-Ethernet (thus limited to a 1 Gbps data rate) and implementing bounded-delay strategies [11]. This technology, due to its compatibility with the regular Ethernet, is oriented toward launchers, manned missions, and space exploration missions, but its applicability for telecommunication and Earth observation missions is now under investigation.

### 1.2. Satellite On-Board Data-Handling Electrical Ground Segment Equipment

Such advancements in terms of data rates require also appropriate control and testing equipment. Electrical Ground Segment Equipment (EGSE) is meant to be used in both the system development phase and system operation. Focusing on system development, proper EGSE shall enable device testing in the least obtrusive way possible. It shall intensively perform a series of tests by injecting a large number of inputs into the target Device Under Test (DUT) (i.e., the payload) while continuously recording, analyzing, saving, and processing the outputs.

**Figure 1:**
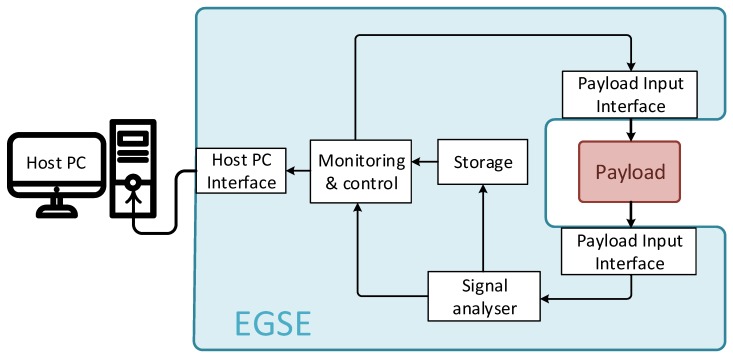
Electrical ground segment equipment generic block scheme.

According to the schema of a generic EGSE shown in Figure 1, this kind of equipment is composed of a monitoring and control block, which is responsible for the generation of both control signals and the input stimuli for the DUT. The DUT outputs are analyzed and, eventually, stored to be used by the control block. There are generally three interfaces: one with the host PC, which controls EGSE operations, and two with the payload (one input, one output). Of course, this is a very abstract conceptual visualization of the system, which may vary significantly, depending on the particular mission requirements.

Ideally, an SPW/SPFI EGSE should be able to have full control, be unobtrusive, and produce and consume data in Real Time (RT). Indeed, RT data generation, consuming, and processing is the most challenging of the capabilities to achieve for this kind of system given the high Gbps throughput requirements.

Current state-of-the-art test and EGSE SPW/SPFI equipment requires system developers and system integrators to rely on many different instruments, in order to address all the following tasks:

- data processing, recording, and playback: to emulate a SpaceWire or SpaceFibre device, which will be interacting with the DUT;
- bandwidth saturation: producing and/or consuming data at a controllable rate;
- unobtrusive monitoring to watch over two nodes without meddling with the link;
- error-injection: to verify corner scenarios either of very circumstantial occurrences (i.e., radiation- induced Bit Error Rate (BER)) or hazardous scenarios for people or property.

EGSE systems can be completely custom designs, or instead, they can be realized on commercial off-the-shelf boards or can even be extensions of general purpose testing systems. Among standard platforms, the Peripheral Component Interconnect (PCI) eXtension for Instrumentation (PXI) is an extension of the PCI specification created to address specifically industry needs for instrumentation and automation in terms of performance and reliability [12]. Modularity is a key feature of PXI based systems, which aims at maximizing integrability and ease of use. One of the most successful applications of this standard is the LabVIEW integration. Indeed, National Instruments is one of the main actors in the PXI standard committee with all its related workflows, such as the National Instruments Hardware In the Loop (NI HIL) platform. Moreover, test equipment is usually tailored for each specific application. Sometimes, their support for the LabVIEW environment or derived workflows is patched up, wrapping driver libraries with all the incompatibility issues and the overhead of the case.

Currently, no solutions based on commercial PXI FPGA peripheral modules for SpaceWire/ SpaceFibre are available [13], and only a few are available for similar protocols [14]. For the SpaceFibre protocol in particular, there are only two pioneering early standard adopter companies offering test equipment: STAR Dundee, with its StarFire Mk3 [15], and the SpaceWire and SpaceFibre Analyser Real Time (SpaceART) from IngeniArs [16]. Both are custom proprietary designs. Furthermore, an early attempt of multi-lane SpaceFibre test equipment was documented in [17]. In this work, a brief comparison between these solution and the proposed one is presented. However, the reader shall consider how the proposed work is the first and only PXI based implementation for LabVIEW. System designers will benefit from the PXI based EGSE: modularity, re-usability, and native LabVIEW support; all features that will lower system development time and costs. The new system based on National Instruments PXI FPGA peripheral modules presented in this paper addresses all the above-mentioned points, for both SpaceWire and SpaceFibre standards. Given the employment of third-party SpaceFibre and SpaceWire IPs and the complexity of a soft-core architecture, a novel cutting-edge LabVIEW FPGA implementation workflow has been followed [18].

This concludes Section 1 of this document, where background knowledge is provided in order to understand the topic and this work’s goals properly.

Section 2 on page 5 discusses in depth the whole system architecture, its hardware/software partitioning, and major design choices.

Section 3 on page 8 provides the reader an accurate report of the challenges to overcome when implementing a complex design into PXI FPGA devices.

Section 4 on page 10 presents the final implementation results in terms of target device resource utilization, along with the verification tests carried out and a noteworthy real use case.

Section 5 on page 14 draws conclusions together with possible speculation on further work’s improvement and impact on the industry. A brief comparison between the proposed system and a major alternative for SPFI equipment on the market is provided.

## 2. Proposed Architecture and Design

**Figure 2:**
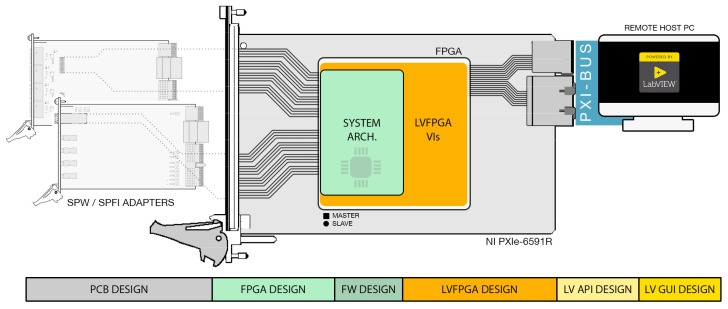
System overall organization and highlight of all encompassed design domains.

**Figure 3:**
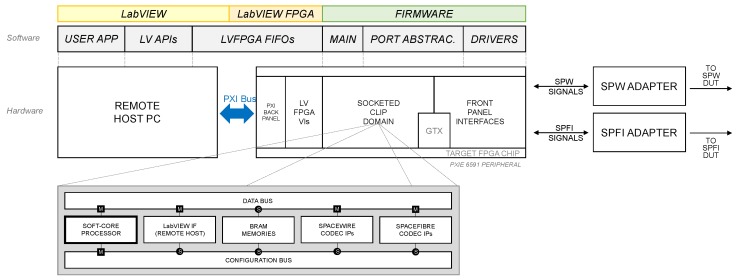
Block diagram of the proposed architecture.

The aim of this work is to offer to the scientific community and the space industry an innovative, proper, and complete instrument for both the SpaceFibre and SpaceWire protocols. This is the first of its kind to be natively supported within the LabVIEW environment for test and measurement at a very high level, yet retaining the finest and deepest controllability and observability over the links. Figure 2 and Figure 3 illustrate the overall system hardware and software architecture, how it is partitioned at a higher level, and of what functional blocks it consists.

The test equipment presented in this paper is new and unique for its kind, because it is endowed with all the following features in one single instrument:

- up to four standard compliant SPW ports;
- up to eight standard compliant SPFI ports;
- eight SPFI Virtual-Channels (VC) per port;
- point-to-point codec interfaces or router IP configurations;
- real-time link status monitoring and control;
- real-time link protocol error report and counter by type;
- SPW/SPFI bridged communication;
- in-hardware packet generation/consumption;
- in-link error injection capabilities;
- word-replacement capabilities over traffic;
- Transmission/Reception (TX/RX) trace memory with triggerable events;
- unobtrusive low level link-analysis monitoring;
- real-time communication with remote-host PC via PXI connection;
- LabVIEW API library package;
- a ready-to-use general purpose LabVIEW Graphical User Interface (GUI) application.

The proposed EGSE solution is organized as a soft-core processor system with dedicated interface hardware accelerators, as shown in the block-diagram at the bottom of Figure 3. The system features multiple buses implementing the Advanced eXtensible Interface (AXI) of the Advanced Microcontroller Bus Architecture (AMBA) 4 specification [19]. Hardware accelerators have a dedicated master interface for DMA concurrent capabilities over the interconnect. The system is completely independent and requires a remote-host PC system running the LabVIEW environment for commands and controls. To add support for the current-generation SpaceWire standard as well seems the most obvious and clear design choice, given the backward compatibility of SPW and SPFI at the packet level.

The system design is rather complex and spans across multiple domains (as highlighted in Figure 2): FPGA design, firmware and drivers’ programming, LabVIEW programming, LabVIEW FPGA programming, and PCB design. hardware/software design partitioning has been decided to maximize performance, avoid any design effort overhead, promote design reuse, and thus, reduce overall development time and costs. All EGSE functionalities and the front-end interfaces are implemented in hardware in order to offer Real-Time RT analysis capabilities and in order to take full advantage of either FPGA on-board resources and previously fully developed Hardware Description Language (HDL) Intellectual Property (IP) cores. Physical PCB adapters are also needed to provide SPW and SPFI standard connectivity. LVFPGA abstracts conveniently the PXI interconnect between the FPGA board and the remote-host PC system into a simpler three-wire handshake protocol. The firmware and drivers are designed to act as coordinator and controller among all system interface ports for concurrent operations. LabVIEW APIs are provided to operate the EGSE system from a remote-host correctly. LabVIEW APIs can be used to design user custom test chains or custom GUI applications. Furthermore, a general-purpose GUI application for Windows hosts is provided together to let the user immediately start using the instrument. This application can control all analysis tools and features of the instrument. It can address most of the test and measurement scenarios on SPW and SPFI links. It is to be highlighted that this GUI application was developed upon the very same LabVIEW APIs mentioned earlier.

### 2.1. Hardware Design

The proposed system implementation is based on the National Instruments PXIe-6591R Peripheral Module (National Instrument, Roscoe, IL, USA). This board has been chosen because it is a commercially available general-purpose FPGA board by National Instruments with LabVIEW native support. It features a Xilinx Kintex-7 410T FPGA unit [20], with on-chip GTX transceivers to implement the SPFI physical layer. This target requires the LabVIEW FPGA module to be programmed.

#### 2.1.1. PCB Design

**Figure 4:**
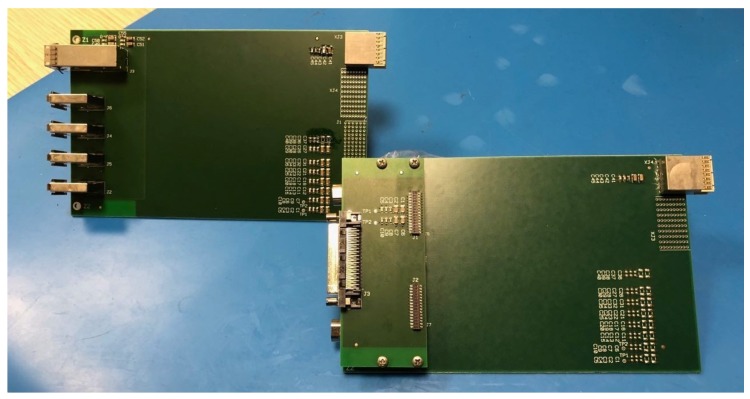
Picture of the adapters for the SpaceFibre and SpaceWire compliant connectors.

The target board has general I/O connectors. To offer instead standard compliant connectivity and connect SPW or SPFI DUTs to the EGSE system, additional adapter modules were designed specifically: the SPFI_eSATA to miniSAS-HD board is a passive physical adapter; the SPW_microD9 to VHDCI-68 board has active buffer components to operate single ended to LVDS signal conversion. The adaptations are bi-directional, and all PCB adapters are made on a FR4 support, sized to fit in the very same host PXI chassis that will host the target NI PXIe-6591 board. The final result is shown in Figure 4. Adapter modules allow expanding the PXIe-6591R VHDCI-68 port to four SPW ports and to convert each miniSAS-HD port into four SPFI ports. Of the 20 single ended I/O signals available on the PXIe-6591R port, 16 were employed for four SPW ports of four single ended data/strobe coded signals each, reserving the four left I/O signals for hardware debugging. The main challenges to overcome for the adapters’ design were careful routed to reduce SPFI signal loss, following best practices like path equalization of differential signals. Given the fragility of such a signal path, using fast-prototyping techniques was simply not viable, and thus, the production was outsourced to a trusted and experienced PCB manufacturing company.

#### 2.1.2. FPGA Design

The hardware architecture implemented in the FPGA target is shown at the bottom of Figure 3 as a block-diagram. It is based on a MicroBlaze™ soft-core processor aided by dedicated interface IPs. Interface IPs are capable of operating DMA transactions on an AXI-4 interconnect. The system is designed as a multiple bus architecture in order to keep conveniently separated interconnect fabrics for TX/RX traffic and configuration data, preventing traffic congestion. This also improves robustness because the MicroBlaze™ processor unit is the only bus master of the configuration bus and, thus, the only one responsible to configure the port interfaces. On-chip block-RAM resources were used as memory buffers in data communications.

The remote host interface through PXI interconnect is supervised by LabVIEW FPGA environment and abstracted as simpler FIFO-like macros with a data-ready-valid handshake protocol. A Direct Memory Access (DMA) capable bridge interface IP was designed specifically in order to translate with minimal latency and maximum throughput such transfers into AMBA 4 AXI burst transactions and initiate them on the system data bus [21].

The front-panel interfaces’ configuration can vary with specific mission needs: SPW point-to-point codec IP, SPW router IP, or SPFI point-to-point codec IP can be instantiated and used as the external port. The only limitation is the number of physically available pins, which limits consequently the number of external ports to a maximum of four SPW and eight SPFI. All SPW and SPFI interface IP cores are derived from currently-flying or flight-ready designs that have been modified in order to obtain as much as possible the observability and controllability over links:

- expose link related statuses and internal signals;
- SPFI/SPW traffic generation/consumption, forwarding, and recording;
- control/data character sniffing and trace back recording;
- low level and high level error injection capabilities (bit flips, control/data characters replacement, error packet termination);
- unobtrusive monitoring of a link between two ports.

One additional feature of this SPFI/SPW test equipment is the interesting possibility to operate a cross-standard communication, since the two protocols are compatible at the packet level [6].

### 2.2. Software Design

Embedded software (also referred to as firmware in the following) is a bare-metal executable created for this system specifically. The design tool of reference is the Software Development Kit (SDK), Version 2018.2, Xilinx, USA in particular. The bare-metal firmware application is executed on the MicroBlaze™ processor instance in order to coordinate the whole system operation. The reasons to opt for a bare-metal solution are essentially three: (1) maximize the performance of the MicroBlaze processor, given its limited resources, (2) minimize the memory footprint, since no dynamic RAM is available within the FPGA, and (3) the operations to carry out are all things considered short and of limited complexity. The firmware architecture is hierarchically organized by consecutive layers of abstraction, as per Figure 2. This encapsulation allows breaking down tasks into simpler steps: the main application executes a general and simple schema for HW initialization and transfer data and control messages following a round-robin scheduling; port and BRAM buffer abstraction offers a general interface to let the main program invoke functional routines; the hardware IP C/C++ drivers underneath carry out HW specific task to achieve the generalized functionality. DMA transfers are handled by exceptions. The abstracted port interface as a more general port class allows updating the system with ease and adding support for more interface protocols in future developments. The abstracted generic ports follow a header-Ack-payload-Ack paradigm with a TX-credit mechanism to operate flow control and avoid bottlenecks. The schema is an original work, and it was conceived to be the most general as possible. Generality is needed in order to cope with a wider range of interface hardware IPs. This paradigm provides the needed flexibility to wrap port interfaces of any hardware kind into a common abstracted interface wrapper. Some hardware may offer some feature intrinsically, and others may not. The features not provided, flow control credit mechanics for example, can be mimicked by means of this paradigm. Unnecessary steps instead can be skipped with dummy functions. Send and receive data transactions are split into two steps: order the transaction, and then, carry out the transaction.

The software architecture also spans the LabVIEW domain in addition to firmware design. End-users can issue commands and configure the system with remote-host LabVIEW APIs or a LabVIEW GUI application. The APIs follow the action engine schema, while the GUI application follows the queued message handler architecture [22].

## 3. PXI Workflow

A key aspect of the proposed system is the design choice to implement the architecture described in Section 2 on a market available FPGA that is natively supported within the LabVIEW environment.

Speaking of the LVFPGA design methodology, it is important to highlight first of all how the LabVIEW FPGA is a tool directed to final users without any Hardware Description Language (HDL) knowledge or skill, who want to benefit anyway from hardware acceleration for their own LabVIEW data processing or test setups [18]. The LVFPGA approach preserves the LV peculiar graphical approach of data flow block designs. HW/SW partitioning is carried out by the tool itself without user control over all the synthesis and implementation processes.

Given that LVFPGA is not addressed to digital HW designers, but final users not fluent in HDL instead, it is a very closed environment in order to reduce human design errors. In particular, the target programmable chip cannot be configured outside the LVFPGA framework. Since reproducing system functionalities as LVFPGA VIs is simply nonviable, a new implementation methodology needs to be defined in order to overcome these limitations.

A special macro function in LVFPGA is intended to import small VHDL sub-designs in an LVFPGA VI, called “Socketed-CLIP”. Despite being carried out within the LVFPGA environment, LVFPGA project VI synthesis and implementation operations fall back to Xilinx tools running in the background (i.e., Vivado for all 7-Series devices). The exploit takes advantage of this dependency: it was found that it is not possible to import in a SckCLIP all kinds of source files that are usually supported in Vivado, yet more than the sole VHDL sources [18] for which the SckCLIP macro is meant. Specifically, the whole hardware HDL design can be imported in a SckCLIP as a single, large, technology dependent EDIF netlist of primitive FPGA resource elements.

**Figure 5:**
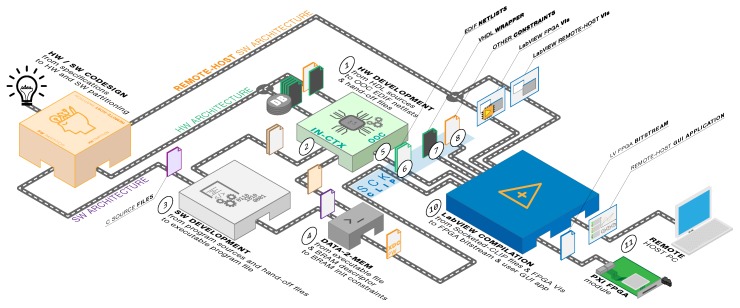
New design workflow exploiting the LVFPGA SckCLIP macro [18].

Figure 5 summarizes the workflow to target National Instruments PXI FPGA peripheral modules taking advantage of the SckCLIP exploit introduced above. FPGA design can be conducted as usual in Vivado with no limitation on source types, but the tool’s support (1), on the workflow diagram shown in Figure 5). Functional simulation can be accomplished as usual as well. A first in-context (in-CTX) synthesis run is carried out (2), necessary to generate block-RAM descriptor and HW hand-off files for SW development. The SW development tool (Xilinx SDK) will compile an executable file for the processor to run as firmware (3). Since there is no means to deploy the generated executable file directly, it is converted into a constraints file to initialize every block-RAM location value according to the generated memory descriptor file (4). An additional Out-Of-Context (OOC) synthesis run shall be carried out (5) to obtain the actual EDIF netlists to be included (6) with its VHDL instantiation wrapper (7) in the upper hierarchy of the whole LVFPGA design. SckCLIP top level ports will be available in LVFPGA as controls and indicators (of the appropriate type). LVFPGA compilation (10) will complete the design implementation, obtaining the bitstream configuration for deployment and remote-host execution (11).

## 4. Results and Use Cases

The proposed EGSE solution implements four SPW point-to-point codec instances and four SPFI point-to-point codec instances. This has been appointed as the reference configuration because it is the most likely configuration to be requested on the market.

The following tests were carried out with an NI PXIe-8880 Controller unit as remote-host PC in an NI PXI-1085 chassis. The instrument was controlled through the provided LabVIEW GUI user application, in order to send/receive data to/from the SpaceFibre and SpaceWire ports, check the test results, and monitor the links in real time, as well as provide higher flexibility in the number of SPFI ports offered.

### 4.1. Results

Implementation results are shown in Table 1. The resource utilization was not negligible; however, no more than 30–40% of the FPGA was occupied, which means that there were resources still available for possible tailored customization. The rightmost columns of Table 1 report indeed the usage per single SPW and SPFI interface respectively in the configuration. The utilization took into account all codec IP resources and all extra resources to provide EGSE functionalities to that IF (for example, the tracing memory core). These data provided a quick estimation of the usage for different configurations in terms of interface number or kind. The EGSE design was indeed modular from that point of view. Router configurations should take into account that codec cores underneath were required anyway.

Table 2 and Table 3 summarize the proposed EGSE system characteristics in comparison with the main design solutions available on the market at the time this document was redacted.

The proposed EGSE system is not only the very first test equipment for SpaceFibre technology natively integrated into LabVIEW. It possesses all analysis capabilities of the most advanced EGSE system available on the market, including unobtrusive monitoring and error injection functionalities. Furthermore, as per Table 2 and Table 3, it is the only instrument capable of offering support up to eight SPFI interfaces and capable of reaching the full bandwidth of SPFI links, up to 6.25 Gbps, at the time this document was redacted. The instrument’s capability of reaching a higher SPW bandwidth, as other market available solutions state they can do, is currently under investigation. The triggering feature is not directly supported in hardware by the PXI FPGA peripheral module, yet can be achieved under the LabVIEW environment, if so programmed. The presented architecture’s high flexibility and modularity allow the better configuration of a proper EGSE setup for each specific space segment to be verified. As a down-side, the PXI setup requires higher hardware costs and initial investments for chassis and controller procurement.

### 4.2. Testing

Various in-field tests were carried out to prove the reliability, strength, and potential of the proposed test equipment.Tests were carried out with the aid of a SpaceART unit, an instrument by IngeniArs.

#### 4.2.1. SPFI Validation Tests

As pointed out, no other test equipment solutions could keep up with the 6.25 Gbps data rate capabilities of the presented EGSE system. Thus, SPFI testing at such high transfer rate was carried out in loop-back configuration. The IngeniArs SpaceART unit was used for 2.5 Gbps tests instead. This also verified that the instrument could correctly communicate with another SPFI device.

A meaningful example of all the extensive tests carried out to validate SPFI capabilities is the tracing memory and error injection the “NACK Test”, where the tracing memory trigger condition was set to catch the first occurring Non-Acknowledgment (NACK) control word. The test aimed to verify the correctness of TX/RX data streaming, either generated/consumed by hardware or by the remote-host, and the effectiveness of the error injection feature as well. The data stream flowed without errors in both directions, and the trigger condition did not occur until the error injection was enabled. Once error injection was activated, the triggering condition was fired, and the dump of tracing memory content clearly showed on screen the traffic data window centered on the triggering NACK word.

#### 4.2.2. SPW Real-Time CRC Calculation and Fly Back

**Figure 6:**
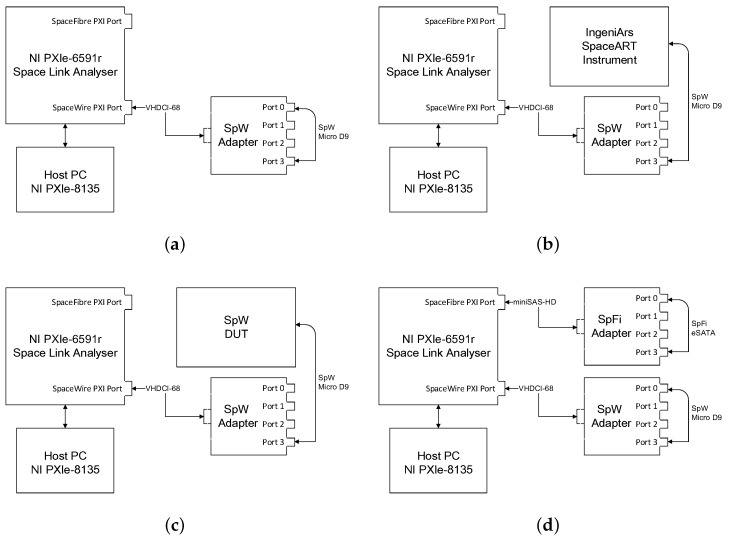
Various setups of the tests carried out. From top to bottom and left to right: (**a**) loop-back configuration, (**b**) SpaceART (IngeniArs) test configuration, (**c**) SpaceWire DUT test configuration, and (**d**) SpaceFibre test configuration.

This test aimed to estimate the latency for which the analyzer system could process and reply. In this test, the system was closed in a loop-back configuration as shown in Figure 6a, transmitting from SPW-Port 1 to SPW-Port 2. The EGSE system was used simultaneously as producer and as consumer with the first SPW port on the SPW adapter connected to the second one. An SPW packet of known size (1000 words of 32 bits)was prepared and sent over the link to the transmission port along with its Cyclic Redundancy Check (CRC) value; once received, the CRC of the packet content was calculated again, compared, and appended. Eventually, the whole packet was sent back. The overall measured latency was 0.4 ms, from the packet dispatch to the packet reception.

#### 4.2.3. SPW Real-Time Full Bandwidth Saturation and Data Logging

The aim of this test was to demonstrate system capability to sustain multiple real-time SpaceWire links without affecting the actual bandwidth. All four SPW links were tested while receiving packets, one at a time. The test setup is shown in Figure 6b. Data were generated with an IngeniArs SpaceART unit, in order to saturate the bandwidth and keep the link under control. Running the test over one hour, the system recorded 67 GB of data on an SSD support. Considering that just 80% of all the SPW transferred bits actually carried information, due to the fact that an SPW data character was ten bits long with a control and a parity bit appended, which means that just eight out of these ten bits contained data [4], we can acknowledge the capability of sustaining a 200 Mbps throughput. Link speed readings from the attached SpaceART unit were congruent.

#### 4.2.4. Device Emulation

In this functional test, an FPGA development board was used in place of flight hardware. A common scenario while developing the spacecraft subsystems is indeed the unavailability of other devices with which to communicate. EGSE systems like the proposed system can act in place of missing devices, replicating the same behavior. Figure 6c shows the direct connection between the instrument and the DUT. Once connected to the unit to be tested, the instrument was capable of letting the user emulate a hypothetical control unit (in this case), preparing telecommand packets, and waiting to receive the telemetry data stream back from flight hardware.

#### 4.2.5. SPFI/SPW Performance Test

This series of functional test was performed with the hardware configuration of Figure 6d. Two SPW ports and two SPFI ports were close in a loop-back configuration. Verifying the SPFI capabilities of the instrument was more complicated because neither the IngeniArs SpaceART nor the StarDundee StarFire MK3 units could cope with the full 6.25 Gbps bandwidth. For this reason, only loop-back tests could be carried out at a 6.25 Gbps link speed.

The SPFI links were tested at different speeds, both 2.5 Gbps and 6.25 Gbps. The FDIR feature of the SpaceFibre protocol was tested and verified. Tests were performed by injecting over the link a wide range of possible Bit Error Rate (BER) values, from 10^−9^ to 10^−5^ values. The SPW links were tested similarly, from 10 to 200 Mbps communication speeds. The error injection feature was verified as well, introducing low-level SPW character errors, bit flips, and replacing the end of packet terminations with the error end of packet.

#### 4.2.6. SPFI/SPW Bridging Test

With the very same test setup of Figure 6d described above, SPFI/SPW cross-protocol compatibility was verified bridging SPW and SPFI data stream. SpaceWire traffic was generated from the first SPW port and received on the second SPW port, then successfully forwarded internally to the first SPFI port on a virtual channel and received on the second SPFI port. No errors, data corruption, nor protocol errors were detected during the performance tests.

### 4.3. Real In-Field Use Case

The presented EGSE system was tested in-the-field in a real case study. The chance to prove its effectiveness and ease of use came from an important company in the aerospace field. Researchers of this company were in need of addressing an unexpected behavior of a device under development. The case scenario was the following: The SPW device after receiving a TeleCommand (TC) at 50 Mbps shall reply with a corresponding TeleMetry (TM) data message at 100 Mbps. TM shall be transmitted without generating bottlenecks on the SPW link. This implies that the receiver SPW node shall consume incoming data assuring that the data rate is sustained in real time. The developers were experiencing sudden and abrupt transmission interruption of the TM stream, but just randomly, once in a while. The developers were using a full custom EGSE solution. Thanks to the new EGSE system presented in this work, it was possible to point out that the device was actually working correctly and that the unexpected disconnections were due to the custom EGSE system: under certain circumstances, the custom EGSE solution in use was not able to consume the stream fast enough, and thus, the device was experiencing overflow.

**Figure 7:**
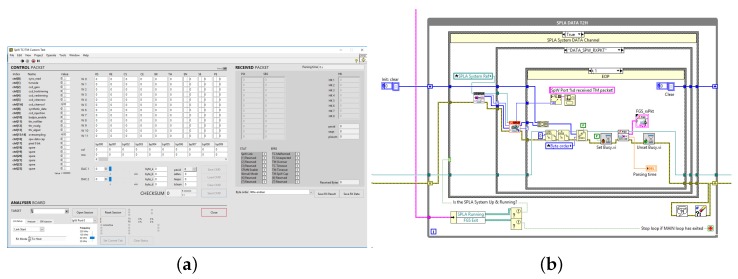
Real Use-Case VI. (**a**) Front-panel view and (**b**) block-diagram view.

Figure 7 shows the custom Test-Chain VI prepared with the provided instrument LV API library, including both the block-diagram and user friendly GUI. Compared to reference test solutions available on the market, the EGSE equipment presented in this paper is natively supported within LabVIEW, thus integrated into the National Instruments environment. This unique characteristic allowed significantly reducing the test setup time, from hardware setup to custom LV test-chain preparation and actual test execution.

## 5. Conclusions

A novel SpaceFibre and SpaceWire test equipment for the LabVIEW environment was presented in this article. The proposed EGSE system was the first SpaceFibre solution to be natively integrated into the LabVIEW environment, thanks to the research in the field of synthesis and implementation methodologies for LVFPGA [18]. Early adopters of the SpaceFibre protocol, as well as experienced SpaceWire developers can benefit from the fine degrees of controllability and observability over the link of interest. The proposed EGSE system is suitable both for on-board device development and spacecraft integration, fulfilling its purposes of offering to the scientific community and to the space industry an innovative and complete equipment for both the SpaceFibre and SpaceWire protocols. All features of the proposed instrument system can be operated with either the provided ready-to-use GUI application or the LabVIEW API library. The system can be configured with up to four SpaceWire ports and up to eight SpaceFibre ports, for point-to-point connections or, for SpaceWire, routers. This modularity allows the EGSE test equipment to replicate as close as possible the real use scenario and to interface with the widest range of spacecraft on-board peripheral, sensors, and other mission specific payload instruments.

This article also provides the reader with a brief survey about the EGSE solutions available on the market for the newly published SpaceFibre protocol, providing a fair comparison in terms of features and performance with the newly developed instrument. From this analysis, SpaceFibre emerged as a very promising technology for the space missions of today and tomorrow, because it addressed data rate requirements in the Gbps order of magnitude.

The EGSE system discussed in these pages is the very first test equipment solution on the market for the SpaceFibre standard implemented on National Instruments COTS PXI FPGA peripheral modules. It is the only current solution capable of operating at SpaceFibre full bandwidth. Its impact on the industry is hard to predict, but hopefully, it will provide the enabling technology in order to promote the diffusion and adoption of the SPFI standard.

## Figures and Tables

**Table 1:** Implementation results on the target NI PXIe-6591R Kintex-7 FPGA, system reference configuration.

Resource	Usage	Availability	Ratio	Each SPW Codec	Each SPFI Codec
Block RAM	420	795	52.83%	32 (4.03%)	40 (5.03%)
LUT	85,380	254,200	33.59%	3752 (1.48%)	11,621 (4.57%)
GTX	4	16	25.00%	0 (0%)	1 (6.25%)
FF Registers	83,644	508,400	16.45%	2416 (0.48%)	8958 (1.76%)

**Table 2:** Comparison table of the available SpaceFibre EGSE solutions by features: maximum bandwidth per link, number of SPW IFs, number of SPFI IFs, type of remote host IF, type of control user experience.

EGSE System	SPFI/SPW Bandwidth	SPW Ports	SPFI Ports	Host IF	User Experience
Proposed SPFI/SPW EGSE	6.25 Gbps/200 Mbps	Up to 4	Up to 8	PXI	LV API or GUI
IngeniArs SpaceART	2.50 Gbps/400 Mbps	4	0 or 2	Ethernet or PCIe	Windows/Linux API or GUI
StarDubdee StarFire MK3	3.20 Gbps/400 Mbps	2	2	USB 3.0	Windows/Linux GUI

**Table 3:** Comparison table of the available SpaceFibre EGSE solutions by capabilities: in-hardware packet generation/consumption, data record, and playback from remote host, SPFI/SPW cross bridging, link error injection, tracing memory, external trigger signals for synchronization.

EGSE System	HW Data Generator/Consumer	Host Reception and Play	SPW/SPFI Bridging	Error Injection	Trace Memory	External Triggers
Proposed SPFI/SPW EGSE	Yes	Yes	Yes	Yes	Yes	No *
IngeniArs SpaceART	Yes	Yes	Yes	Yes	Yes	Yes
StarDundee StarFire MK3	Yes	Yes	Yes	Yes	No	Yes

* Triggering feature can be achieved with the mean of additional signal acquisition PXI Peripheral modules.

## Abbreviations

The following abbreviations are used in this manuscript:

API	Application Programming Interface
BD	Block Diagram
CLIP	Component-Level IP
COTS	Commercial-Off-The-Shelf
CRC	Cyclic Redundancy Check
CTX	Context
DMA	Direct Memory Access
ECSS	European Cooperation for Space Standardization
EDIF	Electronic Design Interchange Format
EGSE	Electrical Ground Segment Equipments
ESA	European Space Agency
FCT	Flow Control Token
FP	Front-Panel
FDIR	Fault Detection Isolation and Recovery
FPGA	Field Programmable Gate Array
GUI	Graphical User Interface
HDL	Hardware Description Language
HW	Hardware
IF	Interface
IP	Intellectual Property
JAXA	Japan Aerospace eXploration Agency
LUT	Look-Up-Tables
LV	LabVIEW
LVDS	Low-Voltage Differential Signaling
LVFPGA	LabVIEW FPGA
MAC	Medium Access Controller
NASA	National Aeronautics and Space Administration
PC	Personal Computer
PCB	Printed Circuit Board
OOC	Out Of Context
QoS	Quality of Service
RAM	Random Access Memory
Reg	Register
RX	Reception, received
SAR	Synthetic Aperture Radars
SckCLIP	Socketed CLIP
SPFI	SpaceFibre
SPW	SpaceWire
SW	Software
TX	Transmission, transmitter
VC	Virtual Channel
VCB	Virtual Channel Buffers
VI	Virtual Instrument
